# Influence of Materials of Moulds and Geometry of Specimens on Mechanical Properties of Grouts Based on Ultrafine Hydraulic Binder

**DOI:** 10.3390/ma17071645

**Published:** 2024-04-03

**Authors:** Beatriz Hortigon, Esperanza Rodriguez-Mayorga, Jose Antonio Santiago-Espinal, Fernando Ancio, Jose Maria Gallardo

**Affiliations:** 1 Departamento de Mecánica de Medios Continuos y Teoría de Estructuras, Escuela Politécnica Superior, Universidad de Sevilla, 41011 Sevilla, Spain; jsespinal@us.es (J.A.S.-E.); ancio@us.es (F.A.); 2 Departamento de Estructuras de Edificación e Ingeniería del Terreno, Escuela Técnica Superior de Arquitectura, Universidad de Sevilla, 41012 Sevilla, Spain; espe@us.es; 3 Departamento de Ingeniería y Ciencia de los Materiales y del Transporte, Escuela Técnica Superior de Ingeniería, Universidad de Sevilla, 41092 Sevilla, Spain; josemar@us.es

**Keywords:** ultrafine hydraulic cement grout, injection of masonry, material of moulds, shape of specimens, compressive strength, flexural strength

## Abstract

Ultrafine hydraulic binder grout injection is a technique utilised for repairing masonry, either to connect sections, seal joints, or fill voids due to its great capacity for penetration and higher mechanical strength than lime grout. In this research, the mechanical properties of ultrafine hydraulic cement grout are analysed considering the influence of the mould material for preparing the specimens and their geometry characteristics in the context of the specifications set out in several international standards. The test campaign to ascertain compressive and flexural strength in different circumstances is supplemented with a physical and chemical characterisation of both binder and fresh and hardened grout. Significant differences in mechanical properties between specimens prepared with absorbent or non-absorbent-water material are found due to the influence of drying shrinkage and decanting binder during the curing process. Furthermore, the slenderness of specimens is presented as an important factor in determining the compressive strength of mixtures.

## 1. Introduction

Grout injections are one of the most common methods for the consolidation of soils. This method has been used for decades in geotechnical applications and always supplies quality results [1,2,3]. Grout injection can be executed in numerous ways, depending mainly on the tool employed to inject and the nature of the soil to be consolidated. A common method of grout injection uses the sleeve-port-pipe as the injection tool, also known by its French name tube-a-manchette (TAM). The procedure is suitable for the consolidation and stabilisation of soils since it enables the injection to be carried out in progressive stages or using reinjection [4]. In this way, in the first stages of the consolidation, binders with larger grain sizes than in the last stages must be used. TAM injections usually begin with cement-based grouts, in many cases coupled with bentonite and different proportions of water. The last stages of grout injected are based on binders with finer grain sizes, such as microfine and ultrafine cements and even chemicals, which provide their greater penetrability, strength, and stiffness.

Grout injection has also long been used as a consolidation technique for ancient masonries. The presence of voids is frequent in old masonries, either due to their own morphology or to the levels of degradation that this historical constructive system presents, thereby rendering it suitable for injection [5,6,7]. These types of masonry are normally injected with lime-based grouts since they are chemically compatible with the usual calcareous nature of ashlars and mortars that can be found in historical buildings. Despite this, several efforts have been focused on modifying the mechanical properties of lime grouts, inherently characterised by low values of strength and stiffness [8]. Admixtures with Portland cement or other additions have also been extensively researched and applied [9,10,11]. It is frequent that these trials use solely Portland cement as the binder. However, in order to attain injectable and masonry-compatible grout, additions are usually included, such as fly ash [12,13,14] and blast-furnace slag [13,15,16]. These additions not only improve the fluidity of the admixtures and reduce bleeding, but they also reduce the carbon footprint of the grouts and are more economical than exclusively cement-based grouts. As a disadvantage, compressive strength, flexural strength, and bond strength decrease, although other additives including pozzolans, such as metakaolin [17,18,19], perlite [20], and bentonite [12], do not affect the mechanical properties of the grouts. Recent interventions have demonstrated that certain types of ultrafine binders perform correctly when applied to historic masonries, such as the interventions in San Dionisio’s Church [21], Santiago’s Church [22,23], and Roman Theatre of Cadiz [24], all of them in Spain.

Full knowledge of the materials used in the repair is compulsory for the success of the intervention. Since ultrafine blast-furnace slag binder grouts are largely used for geotechnical purposes, this must be mechanically characterised when they are injected to repair masonry. This research has been conducted to test and document the values of compressive and flexural strength, along with other physical and chemical properties, of these kinds of grouts.

Significant differences can be found between several standards that refer to grouts regarding the procedure for the preparation of specimens to be tested and their geometry. The American Standard ASTM C1019 [25] deals only with grouts to inject masonries, while the European Standard EN 445 [26] addresses grouts for all purposes. Regarding the material of the moulds, the use of metallic moulds is established in EN 445 [26], and ASTM C1019 [25] offers two possible choices: (i) the use of stony or ceramic moulds in keeping with the masonry to be repaired, or otherwise another absorbent material such as wood, thereby reproducing the grout-masonry bond and (ii) when several specimens are simultaneously manufactured, the lateral faces of the specimens must be moulded with a material similar to masonry units, while the bases of the moulds and the internal partitions between specimens are of a non-absorbent material. The amount of water contained in the mixture is decisive in its final behaviour, and, given the diverse water absorption capacity of said materials, the mould material will be a key factor in the final mechanical properties of the hardened grout. In this context, shrinkage, in that it can induce the appearance of cracks and porosity, is one of the several factors that can affect mechanical properties. Plastic and drying shrinkage must be considered [27]. Plastic shrinkage depends on water loss due to evaporation and water suction from the subbase. Consequently, the use of absorbent moulds must be considered a potential cause of increased shrinkage. On the other hand, drying shrinkage is related to capillary forces as absorbed water is lost in 2.5 to 50 nm pores. Shrinkage increases as the water/binder ratio increases [28] and certain additives, such as blast furnace slag, are incorporated [29].

With regard to obtaining the mechanical properties of the grouts, the standard EN 445 [26] adheres to the mortar standard EN 196-1 [30]. Consequently, most research projects aim to ascertain these properties in accordance with these standards [20,31,32,33], and even standards for concrete are applied [12,14]. Nevertheless, significant differences can be observed in these standards concerning the shape of specimens when ascertaining compressive strength. While ASTM-C1019 [25], EN 196-1 [30], EN 1015-11 [34], and ASTM C942-15 [35] propose a parallelepiped shape, IS 4031-4 [36] establishes cuboid specimens. In this context, several research studies can be found regarding the inversely proportional relationship between the slenderness of concrete [37,38,39] or mortars based on Portland cement [40,41,42] specimens and their compressive strength. 

Specifically, the compressive strength of mortars in joints can also be obtained using the Double Punch Test (hereinafter DPT). The standard DIN 18555-9 [43] proposes thin prism specimens for DPT (Table 1) hardened in moulds of masonry.

Since sealing joints and cracks is one of the main applications of grouts, the results obtained from DPT are considered interesting for the determination of the compression strength of the mixture in this situation. With regard to obtaining flexural strength, the three-point flexural test is the proposed methodology, where researchers [12,31,47,48,49] and standards [30,34,45,50] employ similar shapes and sizes of specimens.

Consequently, this study investigates the effects of the material of the moulds and the shape of the specimens utilised in different standards regarding the testing of grouts made with 0.75 water/binder dosage, based on the flexural and compressive strength and other characteristics of the obtained samples. As an aid to discussing the obtained results, the physical characterisation of the specimens has also been carried out.

## 2. Materials and Methods

This section provides a detailed description of the experimental campaign (Table 2) accomplished to characterise grouts made of microfine ground granulated blast slag (hereinafter GGBS) furnace binders while paying special attention to the specific standard followed in the execution of the test. Hydraulic SPINOR^®^ A12, from HOLCIM S.A:S., Paris, France, has been employed.

### 2.1. Binder

#### 2.1.1. Chemical Composition

According to the product specification, this binder is prepared by finely grinding a mixture of GGBS and clinker. The chemical characterisation provided by the manufacturer (Table 3) gives a typical proportion of the components [51]. This group of binders is usually prepared from 30–70% GGBS and Portland cement mixtures, even though EN 197-1:2011 [52] permits broader ranges. The composition given earlier is an average of the typical compositions of each of the two substances as follows: CaO (64–67%), Al_2_O_3_ (4.4–5.2%), Fe_2_O_3_ (2.2–4.0%), SiO_2_ (21.5–23.5%), MgO (0.7–0.86%) (Clinker), and CaO (30–50%), Al_2_O_3_ (8–24%), SiO_2_ (28–38%), MgO (1–18%) (GGBS).

Scanning Electron Microscopy (SEM) has been carried out in both Zeiss EVO, from Carl Zeiss Microscopy, Jena, Germany, and FEI TENEO equipment, from FEI Company, Hillsboro, OR, USA, for the analysis of cured blocks and received binder, respectively. To this end, a small specimen of binder was adhered to a carbon ribbon and coated with a gold-sputtered film for observation. Morphological and chemical characterisation was carried out using up to 30 kV accelerating voltage and secondary electrons, back-scattered electrons, and OXFORD Energy Dispersive X-ray analysis (hereinafter EDX) detectors, from Oxford Instruments Pic, Abingdon, UK

#### 2.1.2. Particle Size

The binder particle size was measured using a specific dry method for cements, with Malvern Mastersizer 2000 equipment, from Akribis Scientific Limited, Manchester, UK. The Mastersize 2000 measurement technique is based on laser diffraction to find a particular material size distribution. In particular, the equipment measures the intensity of light scattered when the laser beam goes through the sample dispersed in a fluid.

### 2.2. Fresh Grout

Grouts were prepared with a constant dosage water/binder ratio of 0.75, according to both the values recommended for cement-based grouts utilised in the repair of masonry and grouting [53]. A sole additive, superplasticizer Plast 355, from Sika AG, Baar, Swizerland,, was added at a percentage of 5%, in accordance with the manufacturer’s guidelines, to prevent the flocculation of ultrafine particles and the formation of lumps. A Bunsen AGV-10-1321 high-turbulence mixer, from Bunsen S.A., Alcorcon, Spain, with a top speed of 2000 rpm, was employed to prepare the grouts. As a first step, water and the superplasticizer were mixed in a 3 L stainless steel container. Mixing was carried out for 1 min at a low rotary speed of 400 rpm. The binder was subsequently poured as the rotary speed was progressively increased up to 1800 rpm maintaining this value, once was reached, for 15 min. The whole process was carried out at a room temperature of 20 ± 2 °C and a relative humidity of over 50%. Fresh grouts were immediately poured inside the moulds.

**Figure 1:**
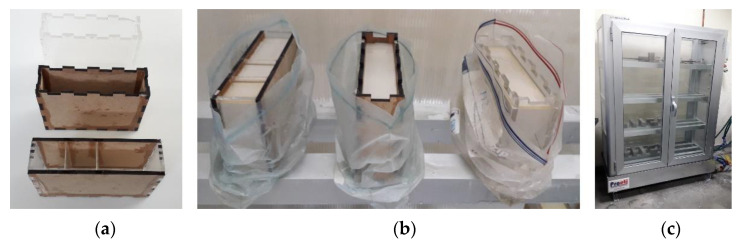
(**a**) type I moulds (methacrylate), type II moulds (MDF), and type III moulds (methacrylate and MDF); (**b**) Curing of fresh grouts inside the curing cabinet; (**c**) Curing cabinet.

In order to consider the influence of the moulds suggested in the standards, two different materials were used: methacrylate as a non-water-absorbent material, in accordance with EN 445 [26]; and medium-density fibreboard (hereinafter MDF) as an absorbent material in accordance with the first choice offered by ASTM C1019 [25]. These materials were combined in three types of moulds. Type I moulds were prepared entirely from methacrylate plates. Type II moulds were made from MDF 10 mm thick boards, and type III moulds combined both materials, also in accordance with [25] in its second choice. Type I and II moulds, measuring 40 × 160 × 110 mm, were utilised to harden blocks individually. In type III moulds, methacrylate and MDF were combined in the lateral walls of the moulds, while internal methacrylate walls divided the internal cavity of the mould into three smaller cavities, two of which measured 40 mm × 40 mm × 110 mm and the third one 40 × 80 × 110 mm (Figure 1a).

The fresh grout, once poured into the moulds, up to a height of 105 mm (Figure 1b), was stored in a curing cabinet at 20 °C and 90% relative humidity for 48 h, at which point they were demoulded. The blocks were then returned to the curing cabinet for 26 additional days maintaining the temperature at 20 °C and the relative humidity at 100% (Figure 1c). In this way, type I, II, and III blocks were obtained for the analysis from the mould type I, II, and III moulds, respectively.

### 2.3. Cured Blocks

#### 2.3.1. Visual Observations/Shrinkage Measurement

Differences between the diverse kinds of blocks after curing can be clearly observed. Density and visual aspects provide evidence of these differences. Visual observations reveal irregular surfaces where the block hardened in contact with MDF (type II and III moulds). More uniform blocks, hardened in contact with methacrylate (type I and III moulds), present certain changes in colour nuances that suggest the decanting of the binder (Table 4).

Another significant difference involves shrinkage, given a common fresh grout initial level of 105 mm. While the final height of type I blocks is 85 mm as a mean value, type II and III moulds lead to blocks with a minimum mean height of 72.5 mm and 72.4 mm, respectively, measured in the centre of the block. Water loss due to mould absorbency or non-absorbency may be assumed as the probable cause of the observed behaviour. Due to the minimum dimensions for the edge of the specimens being established at 40 mm, the top portions of type II and III blocks were discarded, hence coming up with 4 pieces of blocks to analyse: (i) type I-b (bottom area of type I); (ii) type I-t (top area of type I); (iii) type II-b (bottom area of type II) and (iv) type III-b (bottom area of type III) (Table 4).

#### 2.3.2. Physico-Chemical Characterisation by SEM/EDX

SEM and EDX observations of specimens were carried out using microscopes, as described in Section 2.1.1, for the identification of the features that can affect mechanical behaviour, namely porosity, cracks, and second phases. Due to the high hydrophilic nature of the blocks, an ambient pressure SEM at low acceleration voltage had to be used, thereby enabling the characterisation of the block specimens after a thin gold conductive layer had been sputtered over the specimen surface.

#### 2.3.3. Physical Characterisation by Computed Tomography

Computed Tomography (hereinafter CT) was carried out on two small specimens that had been diamond-wheel sawn from inside the bottom part of type I blocks (samples I-b, Table 4). Both specimens were analysed using Yxlon Y.COUGAR.SMT equipment, from Comet Yxlon GmbH, Hamburg, Germany. The first sample was scanned as a small cylindrical volume roughly 1 mm high and 1 mm in diameter using a voxel size of 1.1 µm (sample 1). A second sample, a cylindrical volume of 8 mm high and 8 mm diameter, was scanned with a voxel size of 8.86 µm (sample 2). Image analysis was carried out with free software from Dragonfly (2022.2 non-commercial license).

### 2.4. Specimens

#### 2.4.1. Cut of Specimens

Specimens of a variety of shapes and sizes (Table 5), under different standard specifications (Table 1) were obtained from different areas of the blocks (Table 4). As mentioned in Section 2.3.1, blocks showed several inhomogeneities possibly related to shrinkage and binder decanting. Consequently, certain portions of the blocks had to be discarded (See Table 4 concerning unusable portions). By means of a Wiskehrs M-351-CM cut-off machine, from Wiskehrs Internacional de Elevacion S.A., Zaragoza, Spain, equipped with a 350 mm diameter diamond saw, various types of specimens were obtained (Table 4): (i) type C1 specimens (compression test), which are prisms measuring 40 × 40 × 80 mm, in accordance with EN196-1 [30] and 1015-11 [34], extracted from the bottom and the top of type I blocks (specimens I-C1(b) and I-C1(t)) and from the bottom of type III blocks (III-C1(b)); (ii) type C2 specimens (compression test), which are cubes measuring 40 × 40 × 40 mm. These specimens, following the cubic shape established in IS-4031-4 [44], were cut to the aforementioned dimensions to facilitate comparison with the 40 × 40 × 80 mm specimens described above. Likewise, specimens from the bottom and the top of type I blocks (specimens I-C2(b) and I-C2(t)) and of the bottom of type III blocks (III-C2(b)) were obtained; (iii) type C3 specimens (DPT test), which are prisms measuring 40 × 40 × 16 mm, in accordance with DIN 18555-9 [43]. Furthermore, specimens from the bottom and the top of type I blocks (specimens I-C3(b) and I-C3(t)) and of the bottom of type III blocks (III-C3(b)) were also obtained; and (iv) type F specimens (flexural test): prisms measuring 40 mm × 40 mm × 160 mm, in accordance with EN 196-1 [30]. Specimens were extracted from the bottom and the top of type I blocks (I-F(b) and I-F(t)) and from the bottom of type II blocks (II-F(b)). 

#### 2.4.2. Density

The specimens were weighted in a precision balance in order to measure their density values. Regarding their volume, measurements of dimensions on all the faces of the specimens were carried out and average values of the length of three perpendicular edges were obtained.

#### 2.4.3. Mechanical Testing

The mechanical behaviour of grouts has been successfully characterised by means of compressive tests and flexural tests. According to Section 2.4.1, compressive tests have been carried out on specimens of different shapes and sizes. The variants C1 and C2 are targeted to characterise the compressive behaviour of grouts in standard situations, while C3 is oriented to slender specimens due to the extended use of grouts to seal or to reinforce joints (Table 5). Table 6 shows the design of the experimental campaign in order to characterise the grout under the relevant aforementioned variables.

Mechanical tests were carried out by using a digitally controlled Mohr and Federhaff hydraulic framework (class 1). The maximum load is 100 kN and is equipped with position sensors. The complete set of data obtained from the test was reduced by MATLAB software (v. 9.14) by selecting a reduced cloud of points. To this end, points spaced 0.03% of strain for specimens’ type C1, C2, and F, and 0.05% of strain for specimens’ type C3 were included in the scatter plot.

**Figure 2:**
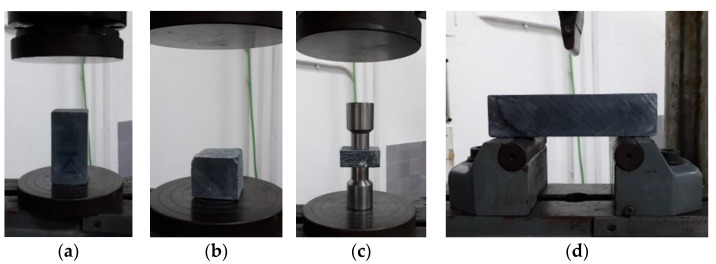
Testing specimens: (**a**) Specimens C1 for the compression test; (**b**) Specimens C2 for the compression test; (**c**) Specimens C3 for the DPT test; (**d**) Specimens F for the flexural test.

Types C1 and C2 specimens were tested in accordance with EN 196-1 [30] to ascertain the compressive strength. A constant testing rate of 200 N/s was used. C3 specimens were tested in accordance with DIN 18555-9 [43] (DPT) (Figure 2).

Compression strength was obtained in these specimens of reduced thickness by using cylindrical punches of 2.5 cm in diameter and a constant testing rate of 200 N/s. The flexural strength of specimens labelled with the prefix F was determined by the three-point flexural test, in accordance with EN 196-1 [30]. In this test, a load is applied in the central section of a horizontal prism at a low rate (10 N/s). The vertical displacement and the load values were registered by the system until failure. A steel cylinder of 10 mm in diameter was employed to support the specimen and to apply the load, respectively. In total, clouds of points ranging from 3521 to 7341 points have been handled to obtain stress–strain laws for these tests as described in the following section. Slenderness of the specimen during the compressive testing can be deduced from Table 5 to be 2 for type C1 specimens; 1, for type C2 specimens; and 0.4, for type CIII specimens. Due to the impossibility of installing strain gauges in the specimens for DPT, the stiffness of specimens has been evaluated by means of the stress–strain rate of each of the tests that have been performed as the slope of the curve at the origin instead of Young’s Modulus.

## 3. Results

### 3.1. Binder

#### 3.1.1. Chemical Composition

Elemental analysis of the received binder powder, at several points and areas, shows the mean results as being Ca (34%), Si (13.9%), Al (4.3%), Mg (2.7%). These values are in agreement with the values deduced from the declared oxide composition by the manufacturer (Ca (34.6%), Si (15.9%), Al (5.5%), Mg (4.3%), except for the magnesium content, which lies below the declared value. An increased clinker content in the binder formula may be responsible for the analysed magnesium content. Moreover, decreasing amounts of S, Fe, K, Na, and Cl have also been detected.

**Figure 3:**
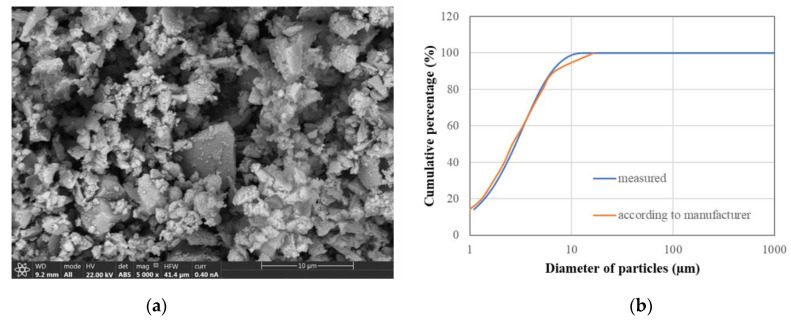
(**a**) SEM back-scattered electrons image of the powdered binder; (**b**) Binder particle size distribution.

As previously discussed, the composition may be consistent with a blend of clinker and blast furnace slag. Moreover, grinding is sufficiently good to render the clinker and slag individual particles hardly distinguishable due to the slight difference expected in their composition, as can be deduced from the uniform grey shades of different particles in Figure 3a.

**Figure 4:**
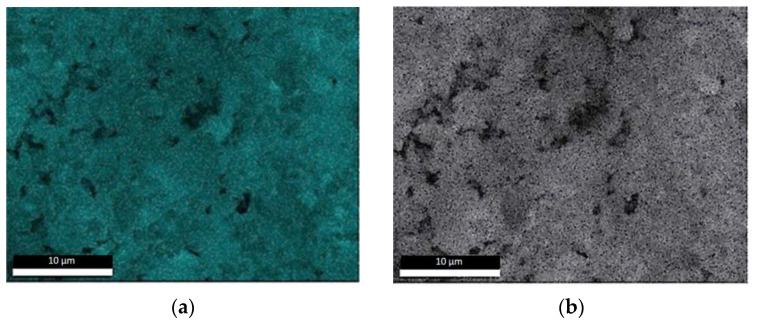

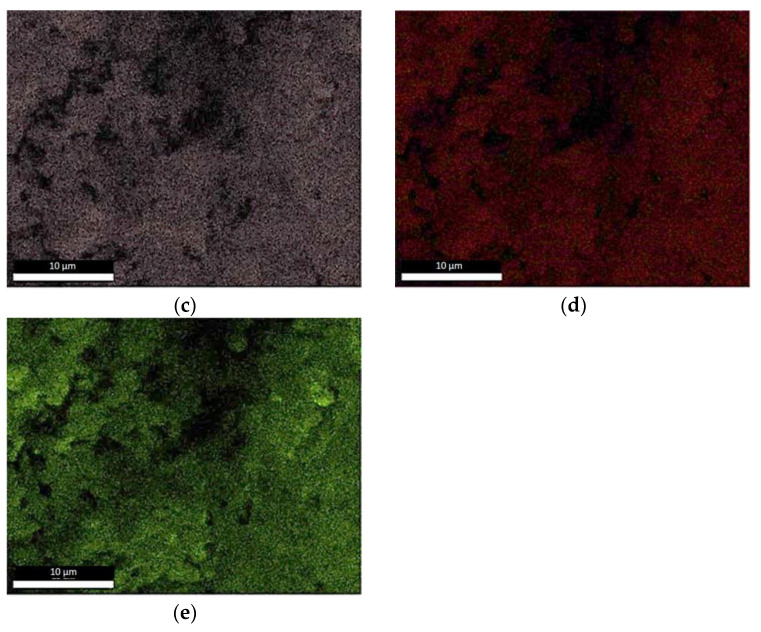
EDX mapping of several elements for the SEM area of Figure 1: (**a**) Calcium; (**b**) Silicon; (**c**) Aluminium; (**d**) Magnesium; (**e**) Oxygen.

Figure 4 corresponds to an elemental chemical EDX mapping of Figure 3a, where brighter areas in each image correspond to a higher content of the represented element. Higher calcium content and lower silica content are expected in clinker particles.

#### 3.1.2. Grain Particle Size

Figure 3b shows binder grain size distribution, which confirms the extremely fine nature of the binder, well suited for use in grout injection projects. According to manufacturer data, the binder grain size is under 12 µm. In fact, actual measurement shows a mere 1.14% of particles over 11.48 µm in diameter while the diameter of 90% of the particles remains under 7.37 µm. By comparing these two distributions, it may be stated that bigger particles are slightly less common than those reported by the manufacturer.

### 3.2. Cured Blocks

#### 3.2.1. Physic-Chemical Characterisation by SEM/EDX

**Figure 5:**
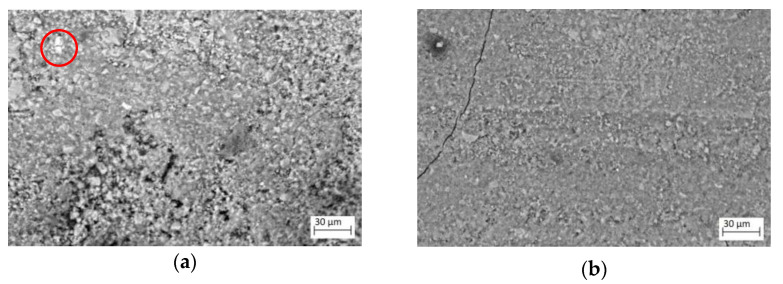

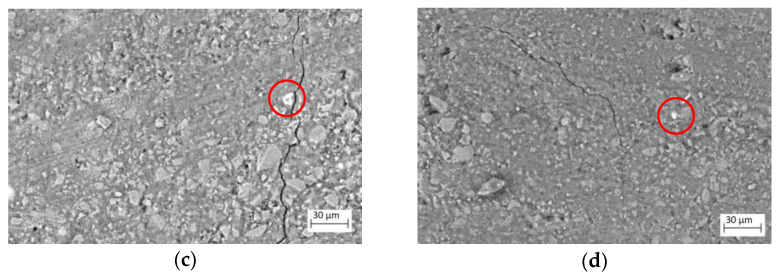
SEM images of sections corresponding to: (**a**) Initial blocks type I-b (methacrylate moulds); (**b**) Initial blocks type I-t (methacrylate moulds); (**c**) Initial blocks type II-b (MDF moulds); (**d**) Initial blocks type III-b (methacrylate-MDF moulds). Inside the red circles, the white phase is associated with an element with a high atomic number (probably iron-rich particles coming from blast-furnace slag).

Figure 5 shows SEM images of the sections taken on several blocks according to the type of mould and the area of the block (top or bottom). Cracking is observed independently of the mould used. Porosity is also observed.

Several phases are present as expected. The figure corresponding to a type I block at the bottom (Initial block I-b) shows one big particle per field (0.2%), while a type I block at the top (Initial block I-t) shows one or two particles 4 µm in diameter per field (<0.1%). type II blocks at the bottom (Initial block II-b) show one or two particles, 5 to 7 µm in diameter per field (<0.1%). Type III blocks at the bottom (Initial block III-b) show 2 to 4 particles, 6 µm in diameter (0.15%). Given that this phase has a particle size of up to 10 µm, it should be identifiable in the CT images, thanks to its higher density and average atomic number in relation to the matrix.

**Figure 6:**
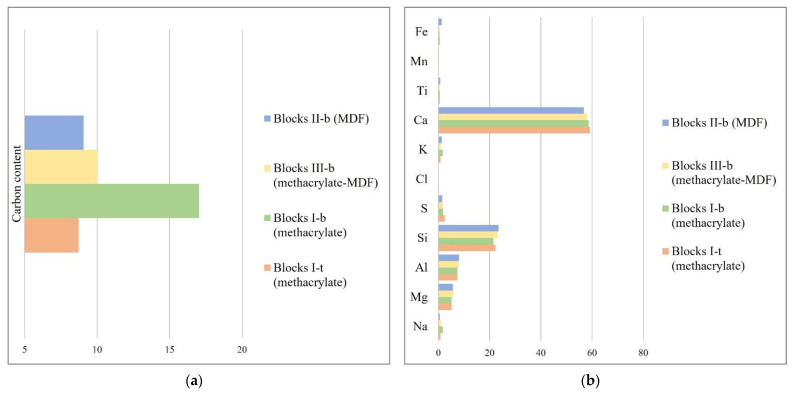
(**a**) Carbon content relative to that of initial blocks type III-b (taken as 10); (**b**) Calcium, Sulphur, Silicon, Aluminium, Magnesium, and Sodium content of specified blocks. Carbon and Oxygen have not been considered in the quantification.

EDX analysis was carried out on the previously presented SEM images. Two measurements at different areas of the blocks were taken. The identified carbon may be related to the added plasticizer, given that both the clinker and the slag are inorganic compounds. The identified amounts of relative carbon are shown in Figure 6a. Results obtained from the bottom of the blocks show a higher carbon content which indicates a superplasticizer decantation. Furthermore, those blocks partially or totally produced from MDF show a certain level of superplasticizer absorption.

On renormalising the compositions measured for the remaining elements (except Carbon and Oxygen, which may be affected by the decantation and absorption of the superplasticizer), it is observed in Figure 6b that the differences between the various blocks and their areas are minimal. The differences observed do not permit clear conclusions to be drawn between the different samples of the three types of moulds used. In fact, the compositional differences are of the order of those found between the two measurement points of the same type of test specimen.

#### 3.2.2. Physic Characterisation by CT

**Figure 7:**
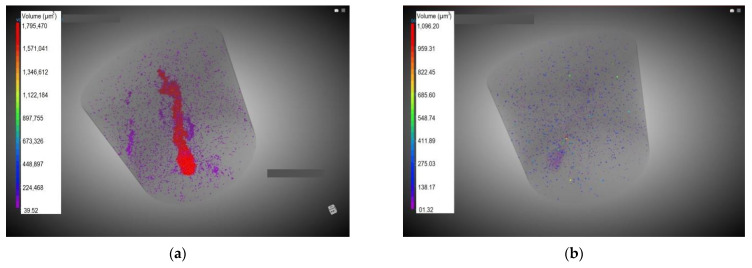
Images obtained from sample 1 CT: (**a**) cracks and pores (colour-coded bar ranges from 39.5 to 1.79 × 10^6^ µm^3^); (**b**) white particles (colour-coded bar ranges from 1.3 to 1096.2 µm^3^).

In the present scan, whose results are shown in Table 7 and Figure 7 for sample 1 and Table 8 for sample 2 (both described in Section 2.3.3), it can be concluded that the mean pore size is of the order of magnitude of binder particle size (Figure 3b).

Regarding sample 1, the total porosity volume was extremely low, at less than 0.1%. White particle content, associated (as previously mentioned in Section 3.2.1) with an element with a high atomic number, probably Fe, is also negligible. These values agree with those found in SEM observations of the samples (Figure 5). While particle distribution is relatively homogeneous (Figure 7a), cracks and pores are clustered in certain areas (grey adjacent areas) (Figure 7b), probably producing mechanically weak zones. Nevertheless, in sample 2, a considerable number of tiny pores detected in sample 1, with a more precise scan, are lost. Consequently, the relative volume of pores and cracks in sample 2 is slightly lower than in sample 1. The same can be stated in connection with the quantification of white particles. The mean diameter of those particles is identified as 61 µm in sample 2 since very small particles with a diameter under 8.86 µm are not detected. Although this method is highly suitable for the detection of tiny pores and particles in the case of the inspected volume is extremely small, in these kinds of intrinsically heterogeneous samples, it may be advisable to scan a larger volume, even if the detection threshold (voxel size) must be increased.

### 3.3. Specimens

#### 3.3.1. Density

Table 9 lists the average values of density measured depending on the area of the initial block type (top or bottom) and on the geometry of the specimen extracted from each of them. The differences found between the densities of the specimens confirm the decantation of the binder observed with the naked eye (Table 4).

Despite all the efforts made to produce homogeneous grouts and considering the probable slow setting speed of the particles (due to their small size), a decantation process has occurred. The terminal velocity for a 12 µm spherical particle in pure water can be computed to be 54 cm/h. That value is indeed an upper limit to the real setting velocities, but small density differences are clear between denser specimens obtained from the bottom compared to those lighter specimens obtained from the top area of type I blocks.

Extracting the specimens from the bottom or the top of type I blocks clearly modifies the results, due to the observed setting of binder in grouts. In this case, the differences in density values are approximately 6% between specimens obtained from the bottom and the top of the block. On the other hand, the density of bottom specimens of type III blocks is higher (1%) than that of type I blocks. Water absorption by MDF will produce a thicker slurry in type II blocks. The same argument is valid in comparing density from bottom specimens from blocks of types I and III.

#### 3.3.2. Mechanical Properties

**Figure 8:**
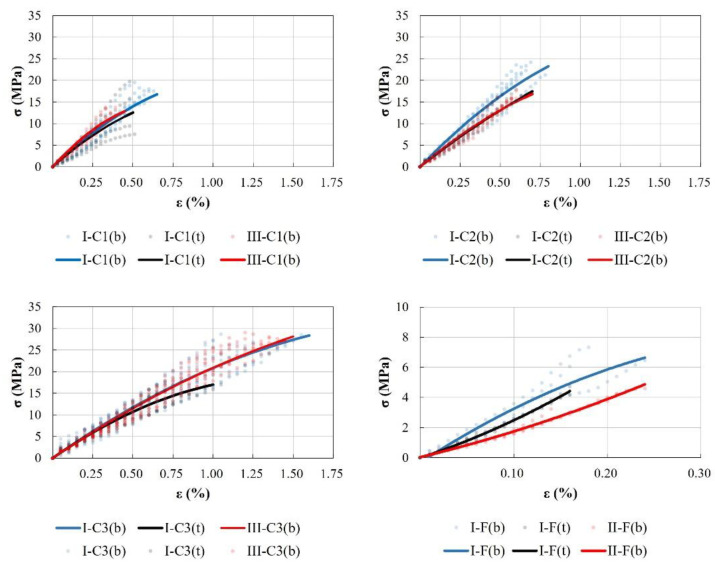
Stress–strain scatter plot and trend lines for the twelve sets of tests, whereby C1, C2, and C3 are tested in compression and F is tested in flexion, according to Table 6.

The results of the mechanical tests are presented in Table 9 and Table 10 and Figure 8. Analysing specimens extracted from type I-b initial blocks, it can be assumed that the differences among the three values of compressive strength (I-C1(b), I-C2(b) and I-C3(b)) are over 27%, and from type I-t initial blocks (I-C1(t), I-C2(t) and I-C3(t)), over 40%. For each geometry, a higher strength is obtained for the denser specimens. Nevertheless, the ratio between density and compressive strength is not proportional for all the specimens, mainly due to the effect of the slenderness of the specimens. Moreover, the effect of the slenderness in the value of the compressive resistance is greater than the effect of the specimen density. Specimen C1 shows, for a given density, the lowest compressive strength, while the effect for shapes C2 and C3 is not as clear, as previously mentioned. Two aspects are worth bearing in mind regarding these differences: (i) slender specimens are more likely to present cracks and defects (observed in CT) which diminish the compressive strength; and (ii) the values of compressive strength for specimens C3 are obtained from a lower area (the area of the contact punch) than those of specimens C1 and C2 (the whole cross-section of the specimens). 

A similar result can be deduced from the stiffness measurements in specimens obtained from type I blocks. In terms of stiffness, the difference between the specimens extracted from the initial blocks type I-b and type I-t reaches 22%, on comparing results of every specimen shape. Again, for every shape (C1, C2, or C3), a higher specimen density means greater stiffness. However, the effect of the specimen shape is greater than that of the specimen density. In this case, for a given density, specimen shape C3 shows the lowest stiffness whereas the effect of shapes CI and C2 remains unclear. Choosing a type I or type III mould produces no significant differences in the results when solely considering density values. Taking the specimens from the bottom part of the blocks as a reference, the difference in the compressive strength lies in the range of 1 to 25% and is lower in type III block specimens compared to type I block specimens. In contrast, the stress–strain rate presents no clear tendency. Moreover, concerning compression strength, density still plays a role when comparing equal specimen shapes. Specimens with the C1 shape attain the lowest values of compressive strength, mainly because of their higher slenderness. However, specimens with the C3 shape present a lower stress–strain ratio than the C1 shape for the specimens prepared with type 1 and type III moulds. These results cannot be explained solely by the water content of the grouts. Type III moulds absorb water, thereby decreasing the water/binder ratio and producing the well-known increase in strength. However, a reduction in strength (1–25%) is observed even though density increases. Regarding flexural strength and density of specimens obtained from type I and type II blocks, greater strength is shown by the denser specimens obtained from the bottom of the type I blocks, as expected. Due to binder particle sedimentation, the water/binder ratio decreases at the bottom of the block, thereby increasing the density and strength. Specimens obtained from type II blocks show even greater densities, due to water absorption by the mould. Nevertheless, the strength is lower than that shown by specimens from type I blocks.

**Figure 9:**
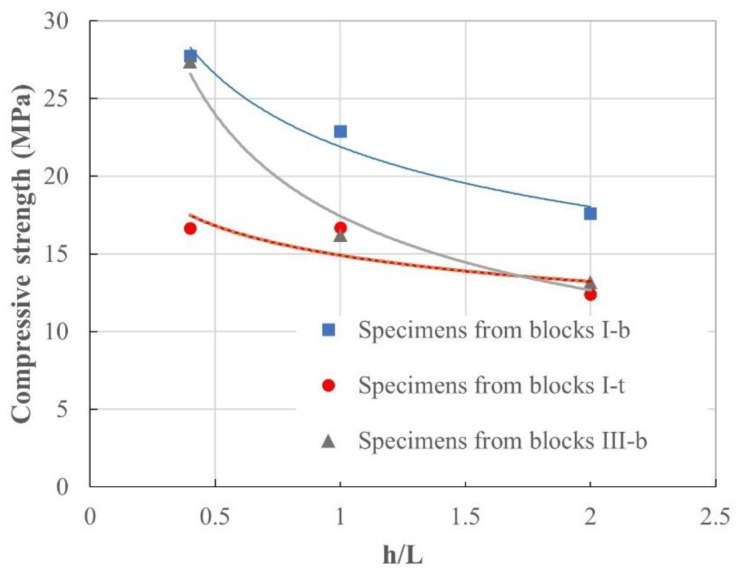
Scatter plot showing the relationship between the compressive strength and the slenderness of specimens obtained from the bottom and the top of initial type I blocks and from the bottom of initial type III blocks.

Regarding the influence of the slenderness of the specimens in compressive strength, a decrease in the strength is evident as the ratio h/L of the specimen increases (Figure 9).

In this way, as slenderness increases from 0.4 to 2.0, compression strength diminishes by up to 50%. A greater trend to a potential curve can be observed in specimens extracted from the bottom of type I and III blocks (I-b and III-b). Specimens extracted from the top of type I blocks (I-t) show a different performance, with a slightly higher compressive strength for specimens I-C2(t) than that of type III-C2(t).

## 4. Conclusions

Grout based on SPINOR A12 binder and concentration water/binder equal to 0.75 has been characterised in detail, from the physical, chemical, and mechanical points of view. In the latter case, the different approaches to the material of moulds and the shape of the specimens set out in several international standards regarding grouts have been taken into account. The majority of these aspects adhere to standards about mortars.

Firstly, this research demonstrates that the physical and chemical characteristics of SPINOR^®^ A12, that is, its grain size and composition, are consistent with the values given by the manufacturer.

The material of moulds affects different aspects of the mechanical results:

1. When absorbent material is used for the moulds, grout shrinkage after setting is greater than when using non-absorbent material. This fact underlines the plastic nature of the shrinkage and also demonstrates the water absorption by moulds.
2. Water absorption by moulds also influences the final values of stiffness and compressive and flexural strength of specimens once the grouts have hardened. All these values are lower when using absorbent materials for moulds than in the opposite case. Overall, high values of density lead to better compression and flexural strength values of the specimens. Nevertheless, in certain cases, high density can be a consequence of a lack of water during setting and, consequently, can lead to the appearance of cracking, which unfavourably affects the mechanical properties of specimens.
3. The decanting of the binder provides different characteristics of specimens obtained from the upper and the lower part of the block manufactured with non-absorbent material in moulds, thereby affecting the results of mechanical tests. Due to binder particle fineness, the expected decantation velocity is sufficiently high to produce lower water/binder ratios at the bottom in the initial stages of setting. This is confirmed by density measurements.

The slenderness of specimens constitutes a key factor in the results of the compression tests. An inverse relationship between this parameter and compressive strength has been observed and quantified.

Therefore, there is a great influence of the mould material when preparing the specimens and their geometry characteristics on the mechanical properties of hydraulic ultrafine cement grout. This is why it is important to consider factors such as the porosity of the masonry to be repaired and the relationship between the expected stresses and the injected mixture thickness before designing the experimental campaign and, consequently, knowing the performance of injected grout.

## Figures and Tables

**Table 1:** Specimen sizes for the compressive test of cement-based mortars and grouts in accordance with different standards.

Standard	Mixture	Country	Specimen Size (mm)	Material of Mould
EN 445 [26] (Refers to EN 196-1 [30])	Grout	Europe	40 × 40 × 80 40 × 40 × 160	Steel
ASTM C942-15 [35]	Grout	United States	40 × 40 × 160	Steel
ASTM C1019 [25]	Grout	United States	Proportions of prism specimen H ^1^ = 2L ^2^	Masonry units with non-absorbent mould bottom and optional spacers
EN 1015-11 [34]	Mortar	Europe	40 × 40 × 80 40 × 40 × 160	Metallic
IS 4031-4 [36]	Mortar	India	70.6 × 70.6 × 70.6	Cast iron/Mild Steel [44]
GB/T 17671 [45]	Mortar	China	40 × 40 × 160	Steel [46]
DIN 18555-9 [43] ^3^	Mortar	Germany	40 × 40 × 16	Masonry units on all the mould faces

^1^ Height of the prism; ^2^ Edge of the base, ^3^ Double-Punch Test.

**Table 2:** Test for the evaluation of grouts.

Analysed Element	Property	Laboratory Test	Section
Binder	Chemical composition	SEM/EDX	Section 2.1.1
	Particle size	Laser diffraction	Section 2.1.2
Cured blocks	Visual observations/Shrinkage	Observation of dimensional changes and cracks	Section 2.3.1
	Physico-chemical characterisation	SEM/EDX	Section 2.3.2
	Physical characterisation	CT	Section 2.3.3
Specimens	Density	Measurement and weight	Section 2.4.2
	Flexural strength	Three-point flexural test	Section 2.4.3
	Compression strength	Compression test/DPT	Section 2.4.3

**Table 3:** Chemical composition (%) of SPINOR^®^ A12.

CaO	Al_2_O_3_	Fe_2_O_3_	SiO_2_	MgO
44	9.5	1.3	31	6.5

**Table 4:** Blocks obtained from moulds of type I, II and III.

Type Block	Mould Material	Visual Aspect	Dimensions (mm)
I	Methacrylate	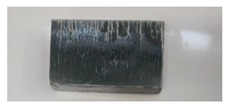	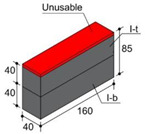
II	MDF	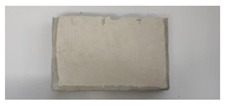	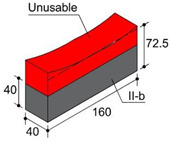
III	Methacrylate-MDF	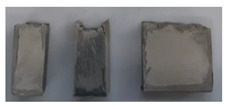	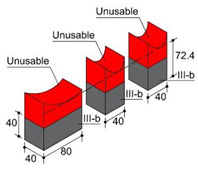

**Table 5:** Quartering of initial sets of blocks in specimens to be tested.

Type of Block	Initial Block	Testing Specimens
Type I (methacrylate)	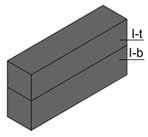	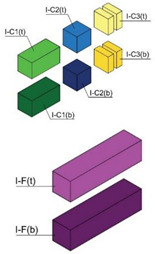
Type II (MDF)	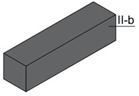	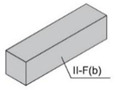
Type III (Methacrylate + MDF)	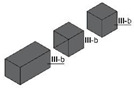	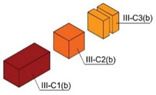

**Table 6:** Types and number of tested specimens.

Type of Mould	Specimen	Block Area	Number of Specimens	Dimensions (mm)	Test
Type I	I-C1(b)	Bottom	5	40 × 40 × 80	Compression as per EN 196-1 [30]
	I-C1(t)	Top			
	I-C2(b)	Bottom	5	40 × 40 × 40	
	I-C2(t)	Top			
	I-C3(b)	Bottom	10	40 × 40 × 16	DPT as per DIN 18555-9 [43]
	I-C3(t)	Top			
	I-F(b)	Bottom	3	40 × 40 × 160	
	I-F(t)	Top			
Type II	II-F(b)	Bottom	3	40 × 40 × 160	Flexural as per EN 196-1 [30]
Type III	III-C1(b)	Bottom	5	40 × 40 × 80	Compression as per EN 196-1 [30]
	III-C2(b)	Bottom	5	40 ×40 × 40	Compression as per EN 196-1 [30]
	III-C3(b)	Bottom	10	40 × 40 × 16	DPT as per DIN 18555-9 [43]

**Table 7:** Quantitative evaluation of CT observations for sample 1 (voxel size 1.2 µm).

Sample 1 ^1^	Number of Features	Mean Diameter (µm)	Sphericity	Total Volume (µm^3^)	Relative Volume (%)
Pores + grey adjacent area	4980	8.1 ± 3.1	0.72 ± 0.10	554,456	0.07
Pores	156,561	2.1 ± 1.2	0.95 ± 0.05	684	9 × 10^−5^
White particles	9982	2.9 ± 2.0	0.95 ± 0.04	188,453	0.02

^1^ Volume tested: 0.76 mm^3^.

**Table 8:** Quantitative evaluation of CT observations for sample 2 (voxel size 8.86 µm).

Sample 2 ^1^	Number of Features	Mean Diameter (µm)	Sphericity	Total Volume (µm^3^ × 10^3^)	Relative Volume (%)
Pores + grey adjacent area	412	215 ± 56	0.32 ± 0.07	213,381	0.05
Pores	40,607	19 ± 16	0.94 ± 0.08	138,777	0.03
White particles	8064	61 ± 20	0.51 ± 0.07	693	2 × 10^−4^

^1^ Volume tested: 403.8 mm^3^.

**Table 9:** Average density, maximum force, compressive/flexural strength (with standard deviation) and stress–strain ratio.

Initial Block	Specimen	Average Density (g/cm^3^)	Compressive/Flexural Strength (MPa)	Stress–Strain Ratio (MPa)
I-b	I-C1(b)	1.836 ± 0.013	17.6 ± 1.8	3141
	I-C2(b)	1.844 ± 0.014	22.9 ± 1.1	3564
	I-C3(b)	1.831 ± 0.029	27.8 ± 1.4	2346
	I-F(b)	1.837 ± 0.023	6.6 ± 0.6	-
I-t	I-C1(t)	1.730 ± 0.015	12.4 ± 6	2972
	I-C2(t)	1.722 ± 0.023	16.7 ± 0.8	2763
	I-C3(t)	1.726 ± 0.027	16.6 ± 0.9	2360
	I-F(t)	1.731 ± 0.012	3.5 ± 1.6	-
II-b	II-F(b)	1.857 ± 0.029	4 ± 0.7	-
III-b	III-C1(b)	1.866 ± 0.053	13.2 ± 0.6	3557
	III-C2(b)	1.856 ± 0.006	16.2 ± 0.8	2892
	III-C3(b)	1.868 ± 0.071	27.4 ± 1.1	2407

**Table 10:** Average density/stress–strain ratio and average density/compressive strength and slenderness for type C specimens.

Initial Block	Specimen	Average Density (g/cm^3^)(×10^2^)/Stress–Strain Ratio (MPa)	Average Density (g/cm^3^)/Compressive Strength (MPa)	Slenderness
I-b	I-C1(b)	0.058	0.10	2
	I-C2(b)	0.052	0.08	1
	I-C3(b)	0.078	0.07	0.4
I-t	I-C1(t)	0.058	0.14	2
	I-C2(t)	0.062	0.10	1
	I-C3(t)	0.073	0.10	0.4
III-b	III-C1(b)	0.052	0.14	2
	III-C2(b)	0.064	0.11	1
	III-C3(b)	0.078	0.07	0.4

## Data Availability

Data are contained within the article.

## Nomenclature

DPT	Double Punch Test
GGBS	Ground Granulated Blast Slag
SEM	Scanning Electron Microscopy
EDX	Energy Dispersive X-ray Analysis
CT	Computed Tomography
MDF	Medium-Density Fibreboard
